# The Institutional Worker

**Published:** 1918-07-06

**Authors:** 


THE
### INSTITUTIONAL WORKER
**Being a Special Supplement to "The Hospital"**

### OUR BUREAU OF INFORMATION.

### Rules for Correspondents.

1. Every letter must be accompanied by the coupon to be cut from the back cover (inside page) of THE HOSPITAL, current issue, and must contain the name and address of the correspondent with pseudonym for publication if desired. Replies by post cannot be given save under exceptional circumstances at the Editor's discretion.
2. Letters from Approved Homes in reply to special needs published in the Bureau should state terms and full particulars, and be sent prepaid under cover to the Editor of the Bureau with name written across coupon for identification.
3. Proprietors of Homes which have not yet been entered on the List of Approved Homes, but have spare accommodation likely to suit special needs, are invited to write for an application form for registration. The fee for registration, which includes two announcements of the Home in the Bureau and other privileges, is 10s.
4. All communications to be addressed to the Editor of THE HOSPITAL, 28 Southampton Street, Strand, London, W.C. 2, and marked "Bureau of Information."

### INSTITUTION FACTS AND FIGURES.

### The Protection of Linen in Bedridden Cases.

#### Answer to May Question.

**By "Thorough."**

THE question was :

**In certain Poor-Law infirmaries ointment and oil mixtures are frequently in use for the treatment of backs and pressure points in bedridden cases, especially when wasting diseases are evidenced. What can be recommended as an economical procedure in the matter of personal and bed linen, bearing in mind the effect of grease and oil on linen ?**

By "bedridden cases," one's thoughts fly to helpless and incontinent cases where very thorough and careful treatment is necessary; and where some form of ointment or skin nourishment is constantly being applied. Zinc ointment is one of the worst for marking bed-linen, leaving a black stain wherever it comes into contact with the linen. Water and air beds will, I presume, be in use to prevent the formation of pressure and bed sores, and with a view to protecting the linen "birds' nests" of tow and absorbent wool can be made and used for any parts where grease is most generously applied. Prevention of marks on linen is better than their cure, therefore I would urge that a *little* grease—and that *little* thoroughly massaged into the skin until it is all absorbed—is infinitely better than much used and left on the skin to soak into the sheets and bed garment. Where ointment or oil dressings are applied the former should be covered by a layer of absorbent wool and the latter by a piece of "protective" and wool; this should form sufficient protection to the bed-linen.

In my training-school, incontinent or bedridden cases showing signs of pressure sores were ordered a preparation of cod-liver oil and malt 3j.T.D.S.A.C., the effect of this internal treatment upon the skin was most marked, and I pass it on as a highly valuable economical hint.

### Restrainer for Diphtheria Case.

#### Answers to June Question.

THE question was :

**Describe a simple, washable restrainer, suitable for helping to keep flat in bed a young child suffering from diphtheria. Such a patient, after the first week or so, does not feel ill, and it is almost impossible to keep a child flat in bed without some kind of restrainer.**

**By "Peter."**

Some strong washable material should be chosen, such as unbleached calico or linen. The restrainer should be made up of double material throughout. An under and an upper piece are required. The under piece should be 10 in. by 15 in. At each side, one inch from top and bottom and two inches from sides, two buttonholes to be worked, 2 in. long, through which the straps of the upper piece are inserted. The upper piece, to consist of a centre portion, should be 11 in. by 8 in., with two straps 2 in. wide at each side. Length of straps varies according to the size of the mattress. Buttonholes are worked at ends of straps for fastening on to buttons sown on the underneath surface of the mattress.

The little patient is placed on the under piece of the restrainer, and the upper piece then adjusted over him. Movement from side to side is thus allowed, but he is prevented from getting into an upright position.

**By "Chad."**

The most simple washable restrainer, suitable for helping to keep flat in bed a young child suffering from diphtheria, is a small sleeveless bodice of calico, fairly tight-fitting, to be worn over the nightgown and fastened at the front with tapes or safety-pins.

At the back of each shoulder of the bodice is a slot three inches in length, and a band of calico two and a half inches in width is passed through these two slots, stretched across the bed, and securely fastened at each side either to the mattress or bed.

This simple restrainer will prevent the child sitting up, but will otherwise allow perfect freedom of arms, legs, and body.

**By E. R.**

A very simple washable restrainer can be made out of three-quarters of a yard of white harding, sufficient to reach from bedhead to over child's chest. Make a fairly large round opening to put the head through easily, and two smaller openings for arms, large enough to allow free arm movements. Bind openings with strong tape and sew on lengths of tape to each corner of restrainer and at bedhead. Tie down to bedstead at the head tightly, and at each side tightly enough to prevent child sitting up and yet allowing ample room for respiration. If the child is allowed a pillow it can be placed quite easily under the head, over the top of the restrainer.

### JULY QUESTIONS.

### 1. War Vacancies.

**Give model "Terms of 'Appointment" applicable for the substitute of a hospital's administrative official who has been called to the Colours. Provision to be made for the possible return to duty of the official previous to the termination of the war and the filling of the appointment in case of the non-return of the official concerned. Describe fully any experiences in this connection that may prove of value to hospital managers generally.**

### 2. Bedroom Suppers for Nurses Off Duty.

**In those hospitals having no kitchen in the Nurses' Home what arrangements can be made whereby the sisters and nurses who are off duty in the evening may have the comfort of supper in their bedrooms? What method may be adopted in order to avoid the dining-room maid being faced by a more or less depleted pantry when she wishes to lay breakfast? (The difficulty seems to be not so much as regards the food as the crockery.)**

#### RULES.

**The following rules must be observed:—**

**1. Contributions must be written on one side of the paper. Brevity and terseness are desirable features. The MSS. must bear the name and address of the sender and be accompanied by coupon to be cut from the back cover (inside page) of the current issue of THE HOSPITAL. A pseudonym must be chosen if the name is not to be published.**

**2. Contributions must be addressed to the Editor of THE HOSPITAL, Institutional Worker Supplement, 28 & 29 Southampton Street, Strand, London, W.C. 2, should reach him before the end of the current month, and be marked in the left-hand corner "Facts and Figures."**

**A minimum payment of Five Shillings will be made for each published answer.**

### QUESTIONS INVITED.

**In connection with our Question Box, we offer every month five shillings each for the two best questions which are sent in for consideration. The questions may be on any institutional subject and concern any department, from the secretary's to the porter's, or the matron's to the domestic staff; the one essential is that they must be practical and deal with points that have a definite relation to hospital or institutional administration, upkeep, finance, or management. Points relating to artificers' work, housekeeping, the laundry, out-patients, and other practical matters will be welcome. It is hoped that thus, when difficult or doubtful points arise in the course of their work, institutional workers will be encouraged to put them in the form of a question and send them to the Editor, THE HOSPITAL Bureau of Information, 28 & 29 Southampton Street, Strand, W.C. 2, marked "Question Box."**

**The best questions will be published in due course and our readers will be given the opportunity of answering them. Thus all institutional workers may be able to cooperate with the view of helping the work and smoothing the difficulties of each other. In short, we want the Question Box of the *Institutional Worker* to be freely used by every worker habitually.**

### ENQUIRIES AND ANSWERS.

### *EMPLOYMENT AND TRAINING.*

### Post Abroad.

WE suggest that you apply to the following: The Colonial Nursing Association, Imperial Institute, S.W.; Lady Minto's Nursing Association, 1 Lindale, Child's Hill, London, N.W.; King Edward Order of Nurses, Kroonstad; the South African Nursing Association, Otto Beit Home, Queen's Road, Johannesburg, South Africa.—JESSIE.

### Certificate of Royal Sanitary Institute.

EXAMINATIONS are held from time to time in Manchester for the certificate of the Royal Sanitary Institute. Write to the Secretary, School of Technology, Sackville Street, Manchester, who will, no doubt, give you full particulars.—MYFANWY.

### *THE HOUSEKEEPERS' DEPARTMENT.*

### Economical Sweet.

MIX together ½ oz. Paisley flour, 2 oz. ordinary flour, 3 oz. caster sugar, and egg powder (amount equivalent to three eggs). Add one tablespoonful of hot water and sufficient milk to mix. Beat all well and lightly together; a quarter of a teaspoonful of vanilla essence is an improvement. Pour the mixture into a prepared square flat baking-tin and bake in a hot oven for about ten minutes. Turn out on to a sugared paper, spread with flour, and roll up and serve hot. The success of this sweet depends upon the beating and baking.—KATH.

### *MISCELLANEOUS.*

### X-Ray Manuals.

A MODERN standard work on radiography and radiotherapy is that by Robert Knox, published by A. and C. Black, Ltd., London. The author has recently published a new edition of his work in two volumes.—X-RAY STUDENT.

"RADIUM, X-rays, and the Living Cell," by Hector A. Colwell, M.B., D.P.H., is published by Messrs. G. Bell and Sons, Ltd., York House, Portugal Street, London, W.C. 2. The price is 12s. 6d. net.—C. L. P.

### Substitutes for Absorbent Cotton.

THE need for conserving absorbent cotton wool used for hospital dressings has led to the introduction of other absorbent materials. Sphagnum moss, to which reference has been made in these columns on several occasions, is used in a great number of hospitals, but still many prejudices obtain against a more general use of this valuable material. It is certainly not so easy to use in dressings on which there is likely to be pressure, such as on the back, but its absorbent properties are remarkable, and there is no doubt that a wider knowledge of this material would lead to its more extensive use. Wood-pulp cellulose has for some time been used by the Central Powers, and has also been introduced into this country. The Americans probably produce this material in the best quality; it is known as cellu-cotton. It is pure white in appearance, of a lacey fineness, and absorbs best when broken up and used in thin layers. Experiments show that it will absorb twenty times its own weight of distilled water. Its capacity is therefore as great as that of the best grade of cotton; it, however, has other advantages, as it holds moisture longer and absorbs more rapidly. We have no doubt that when its use becomes more prevalent and it is manufactured in larger quantities, a considerable saving of money will result from its use.—ECONOMIST.

### The Galvanoset.

WE know of no more suitable apparatus for utilising the current from the main for medical purposes than the "Galvanoset" made by the Medical Supply Association, of Gray's Inn Road, W.C. This ingenious water rheostat can be used with perfect safety for all medical treatments, for the patient's circuit has no metallic contact with the main. Provided that a constant current is available, this apparatus can be utilised for galvanisation and ionisation, and a pleasant undulating and reversing galvanic current can be produced by the simple movement of the controlling device from zero to the maximum required on either pole. With an alternating current any treatment requiring a direct current is not possible. The apparatus is suitable for illuminating all kinds of surgical lamps. There is no other form of rheostat capable of such fine adjustment. The price is £8 8s. Accessory apparatus, including faradic coil and rhythmic interruptor, can be used in connection with it.—P. A. D.

### Aseptic Sputum-Mug.

A STRONG enamelled iron sputum-mug, designed by Dr. S. R. Williams, can be obtained of Messrs. Allen and Hanbury, Ltd., 48 Wigmore Street, London, W. 1. It is inexpensive and easily kept aseptic, as it is not injured by boiling and has no crevices in which the sputum can lodge. The handle of the mug is placed lower down than usual, and from the rim above it a small curved projection passes backward and engages in a small slit in the lid, forming an efficient hinge. The lid is easily removed, and is opened by pressing on its projecting process when raising the cup. The price of one cup is 2s., or 21s. per dozen.—SANATORIUM.

### Address Wanted.

CAN any of our readers give the address of the agent in England who supplies a meat-slicing machine and a butter-cutting machine?—SISTER.

